# The renoprotective effects of tadalafil on ischemia–reperfusion injury during partial nephrectomy in an animal model

**DOI:** 10.1186/s12882-025-04265-2

**Published:** 2025-07-01

**Authors:** Eun Hye Lee, Eun Sang Yoo, Bo Hyun Yoon, Minji Jeon, Tae Gyun Kwon, Bum Soo Kim, Yun-Sok Ha, Man-Hoon Han, Phil Hyun Song, Jae-Wook Chung

**Affiliations:** 1 Joint Institute of Regenerative Medicine, Kyungpook National University, Daegu, Republic of Korea; 2 Department of Urology, School of Medicine, Kyungpook National University, Daegu, Republic of Korea; 3 Department of Pathology, School of Medicine, Kyungpook National University, Daegu, Republic of Korea; 4 Department of Urology, Yeungnam University College of Medicine, Daegu, Republic of Korea

**Keywords:** Partial nephrectomy, Ischemic–reperfusion injury, Tadalafil

## Abstract

**Background:**

Although 25 min is the reported safe partial nephrectomy time for warm ischemia, acute kidney injury occurs even with arterial ligation within 25 min, causing serious complications in patients with chronic renal disease. Various drugs have been studied but evidence of their effectiveness and safety is insufficient. This study investigated the renoprotective function of tadalafil.

**Methods:**

A rat model of partial nephrectomy was treated orally with tadalafil for 14 days before ischemic–reperfusion (IR) injury. Blood and kidney samples were collected for biochemical and molecular analyses 24 h after IR injury. The levels of serum blood urea nitrogen, creatinine, and urine kidney injury molecule-1 were analyzed, while kidney tissues were used for qPCR and histological analysis.

**Results:**

Although effects on blood urea nitrogen and creatine levels were not observed, tadalafil preserved renal function by suppressing the decrease of viable glomeruli, indicating it protected kidneys from IR injury-induced glomeruli loss. Tadalafil effectively reduced the expression of the oxidative stress markers, inducible NOS, endothelial NOS, and myeloperoxidase, and significantly suppressed the expression of inflammation-related genes like TNF-α, IL-1β, IL-6, CD4, and CD8.

**Conclusions:**

Tadalafil inhibits oxidative stress and inflammation, and protects from glomeruli loss during ischemic–reperfusion damage in a rat model of partial nephrectomy.

**Clinical trial number:**

Not applicable.

**Supplementary Information:**

The online version contains supplementary material available at 10.1186/s12882-025-04265-2.

## Introduction

Ischemic–reperfusion (IR) injury, which occurs upon blood flow restriction to an organ followed by blood supply restoration and reoxygenation [1], may be caused by infarction, sepsis, trauma, cardiovascular and urological surgery, shock, or kidney transplantation [2, 3]. The IR injury process includes hypoxia, vascular endothelial injury, inflammatory cell infiltration, free radical accumulation (e.g., reactive oxygen species), and inflammatory chemical generation [4, 5]. IR injury therapies, e.g., machine perfusion, ischemic pre- and post-conditioning, cell therapy, and various pharmacological agents have been investigated in numerous studies [6]. IR injury pathogenesis is unclear and its mechanisms are complex. Although various drugs or compounds, including dapsone, rutin, and eupatilin have been reported to be effective against kidney IR injury [7–9], they have limitations in clinical practice. Therefore, there is a need to research new drugs for minimizing kidney IR injury.

Tadalafil, a phosphodiesterase type-5 (PDE-5) inhibitor widely used to treat erectile dysfunction, increases cyclic guanosine monophosphate (cGMP) levels and blood flow via cGMP’s vasoactive activity [10]. Nitric oxide (NO) is reported to be a key upstream regulator of cGMP signaling [11]. It is synthesized from L-arginine by neuronal, inducible, or endothelial NO synthases and it binds to intracellular soluble guanylyl cyclase, which converts guanosine triphosphate into cGMP [12]. cGMP activates protein kinase G, which modulates ion channels and pumps, thereby reducing calcium influx and promoting intracellular calcium sequestration. By leading to smooth muscle relaxation, this cascade has critical ramifications for vascular tone, endothelial permeability, and cell differentiation [13, 14].

Although tadalafil has been studied in various disorders, including chronic obstructive pulmonary disease [15], benign prostatic hyperplasia [16], pulmonary and systemic hypertension [17], and coronary heart disease [18], the few studies that have investigated its effects on kidney IR [19–21] have applied it to IR injury only. To date, no studies have involved IR injury and volume-reduction injury, such as partial nephrectomy. Here, we investigated tadalafil’s effects on kidney IR injury in a rat model of partial nephrectomy.

## Materials and methods

### Reagents

Antibodies against CD4, CD8, and endothelial nitric oxide synthase (eNOS) were purchased from Abcam (Cambridge, MA, USA). Alexa Fluor 594 (Thermo Fisher Scientific, USA) was used as the secondary antibody. ELISA kits for kidney injury molecule-1 (KIM-1) and myeloperoxidase (MPO) were purchased from Abcam (Cambridge, MA, USA).

### The animal model

All animal study protocols were approved by the institutional animal ethics committee of Yeungnam University, College of Medicine (YUMC-AEC2022-017). A total of 16 six-week-old female Sprague–Dawley rats were purchased from Orient Bio Inc. (Sungnam, Korea). They were then divided randomly into the sham, IR, and IR + Tadalafil groups (*n* = 4, 6, and 6, respectively) and housed in a pathogen-free, temperature-controlled environment with a 12-hour/12-hour light/dark cycle with free access to food and water. The abdomens of the rats in the sham group were opened and closed without nephrectomy. Rats in the IR injury group underwent left kidney removal and right kidney partial nephrectomy. Blood vessel ligation (renal artery and vein) for 30 min was used for IR injury. Before IR injury, the IR injury plus tadalafil group received daily tadalafil (5 mg/kg) for 14 days via oral gavage [5, 22]. Samples were collected 24 h after IR injury. Urine samples, and blood samples for serum blood urea nitrogen (BUN) and creatinine analyses were collected from the metabolic cage and via heart puncture, respectively. The animal inclusion and exclusion criteria were established a priori. Animals were excluded if they had signs of pre-existing illness, abnormal baseline function, or surgical complications like excessive bleeding or infection. Potential confounders like cage location and the order of treatment and measurement were minimized by handling animals in a randomized order during all procedures. All researchers were aware of group allocations. During analysis, data points were excluded if they met the predefined technical error criteria, e.g., instrument malfunction, sample loss, or values falling outside ± 2 standard deviations of the group mean. All criteria were determined before data unblinding.

### Surgical protocol

As per the AVMA guidelines, a two-step method was used to euthanize rats. First, they were gradually anesthetized through the introduction of a 3–5% isoflurane vapor mixed with 100% oxygen into an induction chamber until complete loss of consciousness, followed by their transfer to a euthanasia chamber where CO₂ was slowly introduced at a flow rate of 30–50% chamber volume displacement per minute, until breathing cessation, pupil dilation, and lack of movement. Both kidneys were exposed by making a dorsal slit. A bulldog clamp was used to ligate the left kidney, and renal vessels were tied off using a black silk suture before completely removing the kidney. The right was clamped using a bulldog clamp and subjected to ischemia for 30 min to induce IR injury. Additionally, the right kidney’s middle pole (approximately 3/4 of a kidney) was removed surgically. Hemostasis was achieved using a 100% pure natural collagen agent (Novacol fibrillar^®^). After clamp removal, the kidney was carefully inspected for bleeding and repositioned in the abdominal cavity, followed by peritoneum and skin closure using interrupted sutures. Hypothermia was prevented by placing the animals on a warming pad or heating blanket immediately after surgery and maintaining the body temperature at 37 ± 1 °C. Postoperative pain was managed by intraperitoneally administering meloxicam (1–2 mg/kg). Animal movement, posture, surgical wound condition, signs of infection, and body weight were monitored daily for at least 1–3 days after surgery. To ensure proper wound healing and prevent infection, surgical sites were checked regularly and disinfected as needed. After kidney tissue sampling, death was confirmed through cervical dislocation.

### Hematoxylin and Eosin (H&E) staining

Kidney tissues were immediately fixed in 4% formalin, dehydrated in a gradual ethanol solution, and embedded in paraffin. They were then sectioned at a thickness of 4 μm, placed on charged glass slides, deparaffinized, rehydrated in gradual ethanol, subjected to H&E staining, mounted, and examined under a light microscope. Tubule injury scoring was assessed on H&E-stained tissue sections based on the criteria described in Table 1.

**Table 1:** Tubule injury score

Grade	Injury	Pathology description
0	None	Normal tubule
1	Slight	Mild blebbing, loss of brush
2	Mild	Intensive blebbing, mild vacuolization
3	Moderate	Shrunken nuclei, intensive vacuolization
4	Severe	Necrotic/apoptotic cells Basement membrane denudation/rupture
5	Necrosis	Total tubule necrosis

### Quantitative real-time PCR

After sampling, kidney tissues were immediately stored at -80 °C. RNA was extracted using a Maxwell^®^ RSC simply RNA Cells Kit and a Maxwell™ instrument (Promega, Madison, WI, USA). After RNA quantification, RNA (1 ug) was reversed transcribed into cDNA using GoScript™ Reverse Transcriptase (Promega, Madison, WI, USA). A StepOnePlus™ Real-Time PCR System (Applied Biosystems^®^ Inc., Foster City, CA, USA) was used for quantitative real-time PCR using the primers in Table 2 and the following program: initial denaturation at 95 °C for one minute, followed by 38 cycles of denaturation at 95 °C for three seconds and combined annealing/extension at 60 °C for 30 s. Fold-change gene expression was calculated using the 2^-ΔΔCt method and GAPDH as the reference gene. First, the ΔCt value was calculated by subtracting the Ct value of GAPDH from that of the target gene (ΔCt = Ct_target - Ct_GAPDH). ΔΔCt was then determined by subtracting the ΔCt of the control group (calibrator) from that of the experimental group (ΔΔCt = ΔCt_experimental - ΔCt_control). Finally, fold-change was calculated using the formula, 2^-ΔΔCt, with values of > 1 and < 1 indicating upregulation and downregulation, respectively, relative to the control group.

**Table 2:** Primer sequences

Gene	Forward (5’–3’)	Reverse (5’–3’)
TNF-α	ACACACGAGACGCTGAAGTA	GGAACAGTCTGGGAAGCTCT
IL-1β	GGGATGATGACGACCTGCTA	TGTCGTTGCTTGTCTCTCCT
IL-6	CTCATTCTGTCTCGAGCCCA	CTGTGAAGTCTCCTCTCCGG
iNOS	CCTTCCCTCCCGTTTTCTCT	GTTGGGAGTGGACGAAGGTA
GAPDH	CATCACTGCCACCCAGAAGACTG	ATGCCAGTGAGCTTCCCGTTCAG

### Immunofluorescence

Tissues were fixed in 10% formalin, embedded in paraffin, sectioned at a 4-µm thickness, and placed on charged glass slides. The slides were then incubated in citrate buffer for antigen retrieval after hydration. Tissues were then incubated in a blocking solution, and then with the primary antibodies against eNOS, CD4, CD8, MPO, F4/80, HLA-DR (at 1:100) at 4 °C overnight. The slides were then mounted using an aqueous mounting medium containing DAPI (Vector, Burlingame, CA) and examined under a fluorescence microscope.

### Statistical analyses

Differences between multiple groups were compared using one-way ANOVA. Post hoc comparisons were conducted using Tukey’s multiple comparison test when a significant main effect was observed. Data are presented as mean ± standard deviation (SD). A p-value of < 0.05 was considered statistically significant.

## Results

### Tadalafil’s effect on kidney function

**Fig. 1:**
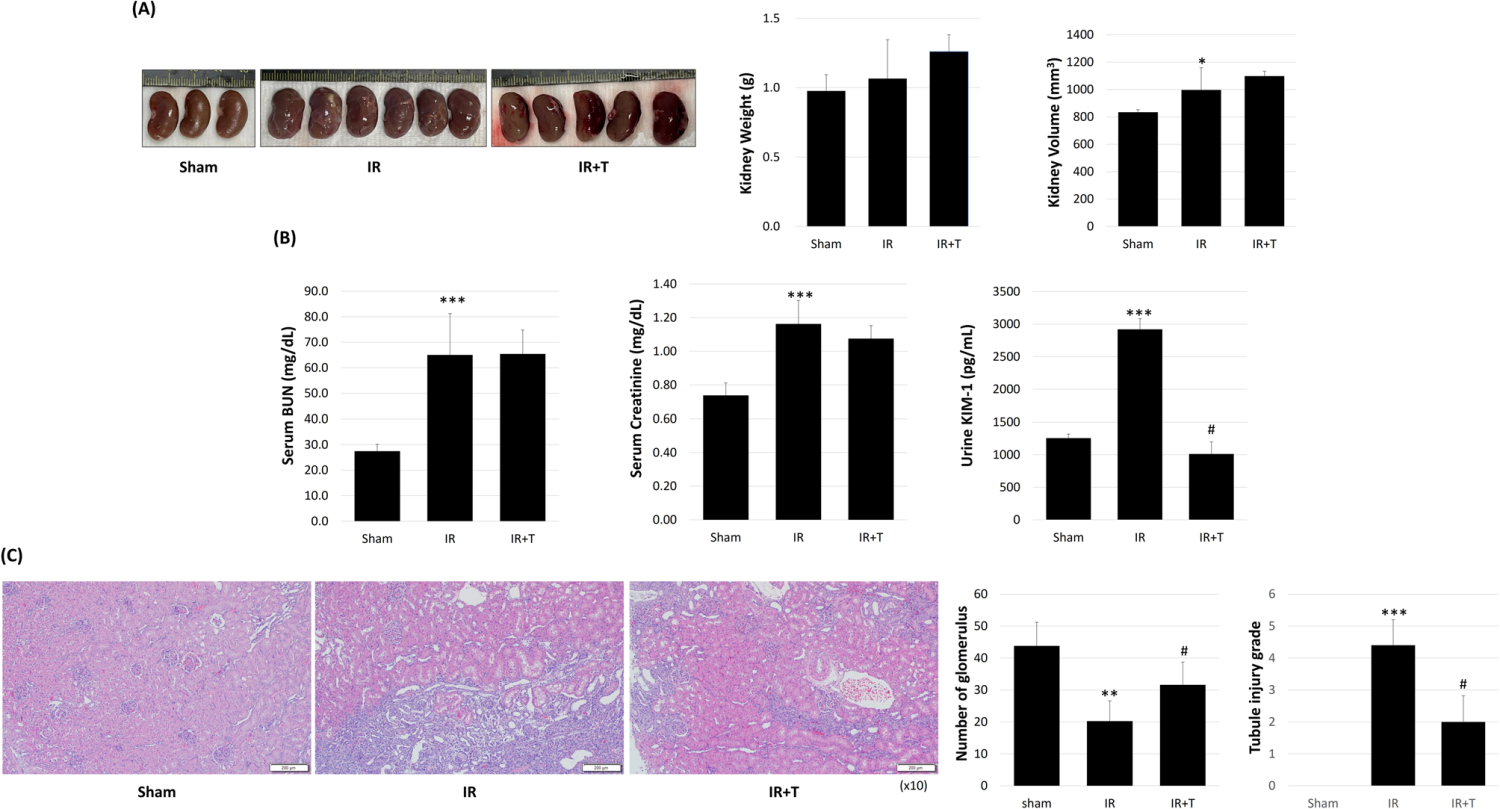
Gross image and serum and tissue analysis. (**A**) Kidney gross image, weight, and volume. (**B**) Serum and urine chemistry analysis. (**C**) Representative H&E staining image, glomeruli number, and tubule injury scoring. Data represent the mean ± SD of three independent experiments (*, **, and *** indicate *p* < 0.05, < 0.01, and < 0.001, respectively, between the sham and IR groups. #, ##, and ### indicate *p* < 0.05, < 0.01, and < 0.001, respectively, between the IR and IR + T groups. *N* = 4–6 per group

A comparison of tadalafil’s effect on kidney weight and volume when compared with the sham group revealed elevated weight in the IR and IR + T groups (0.98 ± 0.11 g vs. 1.06 ± 0.28 g vs. 1.26 ± 0.12 g), although the difference was not significant (*p* = 0.536 and 0.199). Compared with the sham group, tissue volume was increased in the IR group (840.12 ± 22.48 mm^3^ vs. 1046.31 ± 164.41 mm^3^, *p* = 0.025), but it was not significantly changed in the IR and IR + T groups (1046.31 ± 164.41 mm^3^ vs. 1087.71 ± 26.92mm^3^, *p* = 0.591; Fig. 1A).

Tadalafil’s effect on kidney function was evaluated by analyzing the levels of serum BUN, serum creatinine, and urine KIM-1. Compared with the sham group, serum BUN was highly increased in the IR group (27.5 ± 2.7 mg/dL vs. 65 ± 16.2 mg/dL, *p* < 0.01). However, tadalafil did not inhibit serum BUN elevation in the IR + T group (65 ± 16.2 mg/dL vs. 65.4 ± 9.4 mg/dL, *p* = 0.97). Compared with the sham group, serum creatinine levels were elevated in the IR group, and they were not decreased by tadalafil (0.74 ± 0.07 mg/dL vs. 1.16 ± 0.14 mg/dL vs. 1.08 ± 0.08 mg/dL, *p* = 0.0006 and 0.315). Treatment with tadalafil effectively suppressed urine KIM-1 elevation in the IR + T group when compared with the IR group (1255.067 ± 61.28 pg/mL vs. 2919.733 ± 162.16 pg/mL vs. 1010.4 ± 186.67 pg/mL, *p* = 0.005 and 0.008; Fig. 1B).

Kidney microanatomy H&E analysis revealed that compared with the sham group, IR injury decreased glomeruli number and that tadalafil preserved renal function by significantly suppressing the decrease in viable glomeruli (43.8 ± 7.32 vs. 20.25 ± 6.34 vs. 31.6 ± 7.09, *p* = 0.001 and 0.041; Fig. 1C). This is a result of less glomeruli damage and a lower number of IR injury-affected glomeruli when compared with the group without tadalafil treatment. Additionally, a professional pathologist evaluated the level of renal tubule injury, an important characteristic of acute kidney injury, and observed a significantly increased tubule injury score in the IR group when compared with the sham group, and a significantly reduced score in the IR + T group (0.00 ± 0.00 vs. 4.4 ± 0.80 vs. 2.0 ± 0.82, *p* = 0.000 and 0.012; Fig. 1C).

### Tadalafil’s effect on oxidative stress

**Fig. 2:**
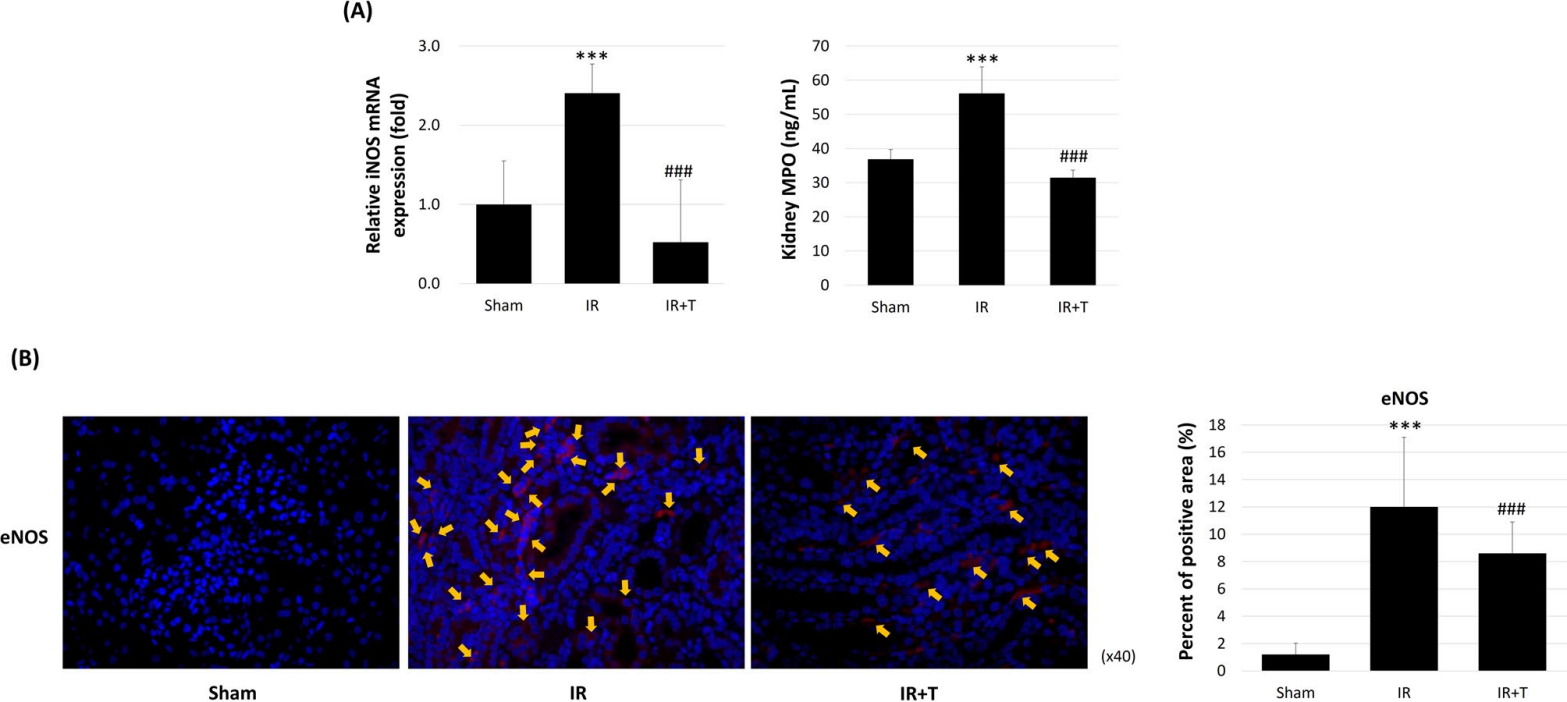
Tadalafil’s effect on oxidative stress. (**A**) iNOS and kidney MPO expression levels. (**B**) eNOS immunofluorescence staining. Data represent the mean ± SD of three independent experiments. *, **, and *** indicate *p* < 0.05, < 0.01, and < 0.001, respectively, between the sham and IR groups. #, ##, and ### indicate *p* < 0.05, < 0.01, and < 0.001, respectively, between the IR and IR + T groups. *N* = 4–6 per group

An evaluation of tadalafil’s antioxidant effects in kidney tissues revealed inducible nitric oxide synthase (iNOS) elevation in the IR group when compared with the sham group, and that tadalafil protected from iNOS increase (1 ± 0.55-fold vs. 2.41 ± 0.37-fold vs. 0.52 ± 0.79-fold, *p* = 0.0000 and 0.0001). IR injury increased MPO levels, which were suppressed by tadalafil (36.86 ± 2.82 ng/mL vs. 56.12 ± 7.75 ng/mL vs. 31.47 ± 2.21 ng/mL, *p* = 0.0004 and 0.0008; Fig. 2A). Immunofluorescence staining revealed a higher number of eNOS-positive cells in the IR group when compared with the IR + T group (Fig. 2B). Quantitative analysis of eNOS immunofluorescence staining revealed a significantly increased positive area in the IR group when compared with the sham group, while the eNOS-positive area was reduced in the IR + T group (1.20 ± 0.83 vs. 12.00 ± 5.09 vs. 8.60 ± 2.54, *p* = 0.003 and 0.001; Fig. 2B).

### Tadalafil’s effects on kidney inflammation

**Fig. 3:**
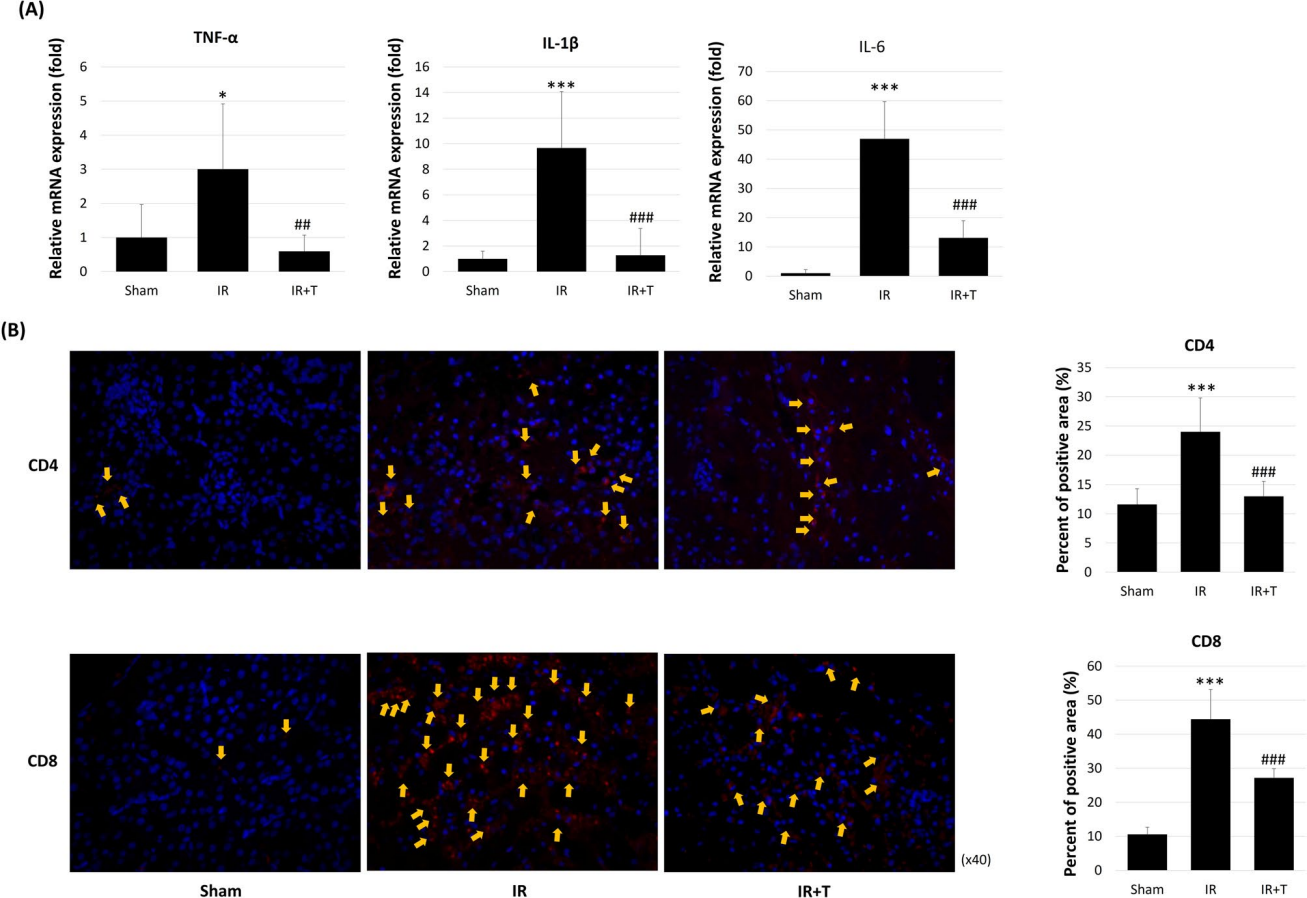

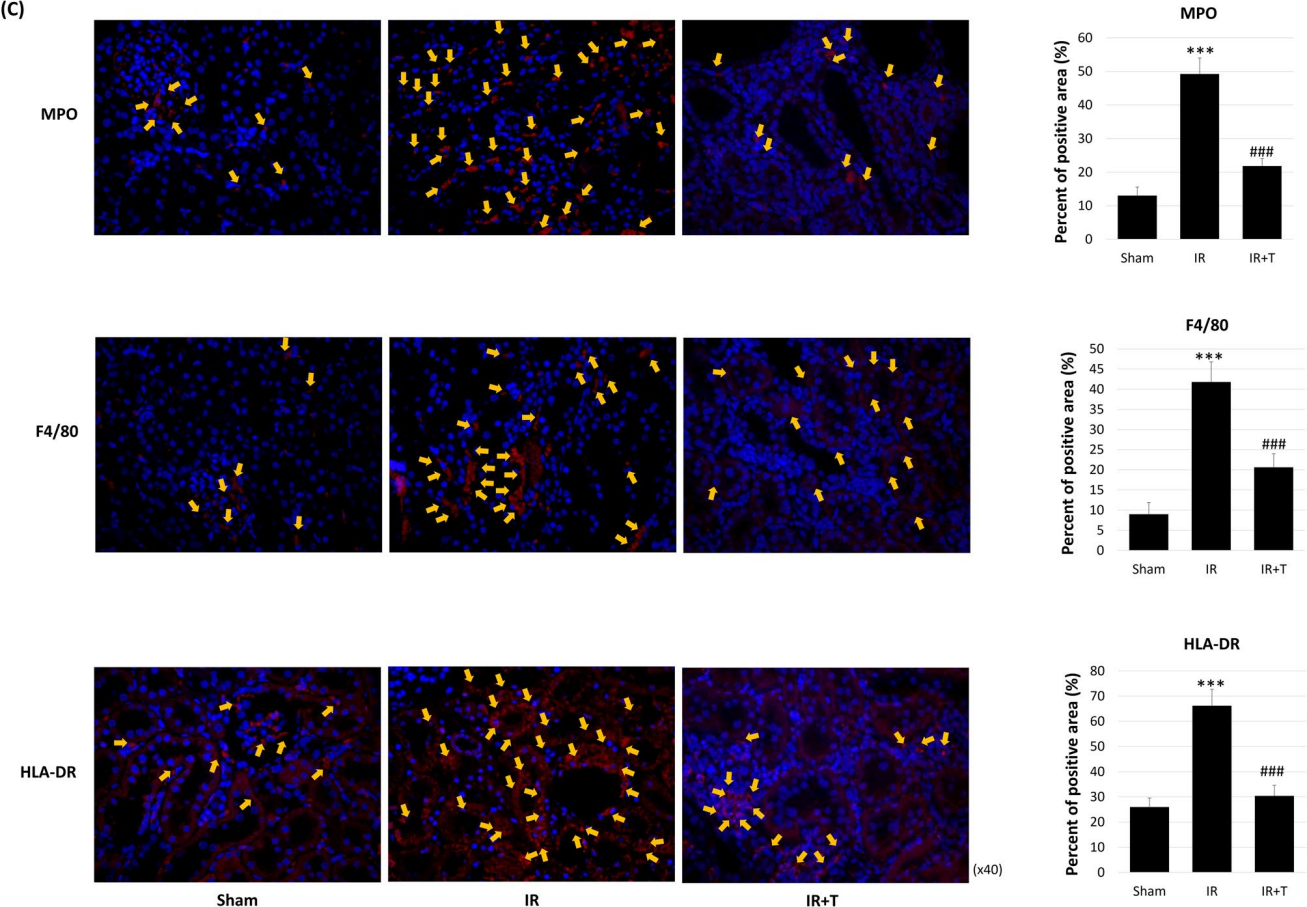
Tadalafil’s effects on inflammation and lymphocyte recruitment. (**A**) Quantitative real-time PCR analysis of inflammation-related gene expression. (**B**) Immunofluorescence detection of CD4- and CD8-positive lymphocytes. (**C**) Immunofluorescence detection of neutrophils (MPO), macrophages (F4/80), and monocytes (HLA-DR). Data represent the mean ± SD of three independent experiments. *, **, and *** indicate *p* < 0.05, < 0.01, and < 0.001, respectively, between the sham and IR groups. #, ##, and ###, indicate *p* < 0.05, < 0.01, and < 0.001, respectively, between the IR and IR + T groups. *N* = 4–6 per group

An investigation of tadalafil’s effect on inflammation revealed that TNF-α, IL-1β, and IL-6 were elevated in the IR group when compared with the sham group, and decreased in the IR + T group ([1 ± 0.96-fold vs. 3.00 ± 1.91-fold vs. 0.59 ± 0.47-fold, *p* = 0.243 and 0.004], [1 ± 0.58-fold vs. 9.65 ± 4.42-fold vs. 1.28 ± 2.09-fold, *p* < 0.0000 and = 0.0009], [1 ± 1.04-fold vs. 46.96 ± 12.70-fold vs. 13.08 ± 5.84, *p* < 0.0000 and 0.0000], respectively; Fig. 3A). Lymphocyte recruitment analysis by staining revealed that as expected, CD4- and CD8-positive cells were increased in the IR group, while the IR + T group exhibited fewer positive cells (Fig. 3B). CD4 and CD8 immunofluorescence-positive areas were markedly increased in the IR group when compared with the sham group, while the IR + T group exhibited a significant reduction. The CD4 positive areas were 11.6 ± 2.70, 24.00 ± 5.78, and 13.00 ± 2.54 in the sham, IR, and IR + T groups, respectively (*p* = 0.002 and 0.004; Fig. 3B). For CD8, the corresponding values were 10.6 ± 2.07, 44.4 ± 8.73, and 27.2 ± 2.68, respectively (*p* = 0.000 and 0.002; Fig. 3B). Next, we analyzed MPO, F4/80, and HLA-DR to detect neutrophils, macrophages, and monocytes, respectively (Fig. 3C). As expected, these proteins were elevated in the IR group when compared with the sham group, while their expression was decreased in the IR + T group when compared with the IR group. Immunofluorescence revealed that the MPO, F4/80, and HLA-DR positive areas were markedly increased in the IR group when compared with the sham group, and significantly reduced in the IR + T group. MPO-positive areas were 13.0 ± 2.55, 49.2 ± 4.76, and 21.8 ± 2.28 in the sham, IR, and IR + T groups, respectively. For F4/80, the values were 9.0 ± 2.92, 41.8 ± 4.97, and 20.6 ± 3.36, respectively. For HLA-DR, the corresponding values were 26.0 ± 3.54, 66.2 ± 6.57, and 30.4 ± 4.22 (Fig. 3C).

## Discussion

Tadalafil, a PDE-5 inhibitor, is the gold standard for erectile dysfunction treatment. PDE-5 inhibitors are effective against inflammation, pulmonary hypertension, esophageal motility disorders, and diabetes [23–26]. Although it was originally developed for angina pectoris treatment, evidence suggests that tadalafil has broad inhibitory effects on the breakdown of cGMP, the second messenger for NO and natriuretic peptides, resulting in vasodilation [27]. Additionally, it has effects against head and neck squamous cell carcinoma, colorectal cancer, prostate cancer, and hepatocellular carcinoma [28–31]. Here, we show that by reducing inflammation and alleviating antioxidative stress, tadalafil has potential renoprotective effects.

Our findings demonstrate that tadalafil exhibits renoprotective effects against IR injury in a rat model of partial nephrectomy. Although previous studies have reported on the effects of therapy on an IR injury model only, our study demonstrates tadalafil’s renoprotective effects by combining IR injury with partial nephrectomy, i.e., using a model of direct renal tissue volume loss.

KIM-1, a type I transmembrane glycoprotein, is a promising kidney injury biomarker [32]. The levels of urine KIM-1 increase rapidly upon tubular injury and are associated with tubular injury degree, interstitial fibrosis, and inflammation in ischemia-, hypoxia-, or toxicity-damaged kidneys [33]. Tadalafil prevented urine KIM-1 elevation without effectively decreasing serum BUN and creatinine levels.

Although serum creatinine was not significantly decreased in this study, significant results were observed for glomeruli, which filter plasma and initiate urine formation, the main kidney function [34]. Ischemic injury decreased glomeruli number in the IR group and tadalafil significantly protected viable glomeruli from IR injury. PDE5 inhibition in the kidney increases NO levels, a deficit associated with acute chronic kidney pathophysiology [35]. Tadalafil and sildenafil selectively inhibit PDE5 binding to cGMP. Since cGMP is a NO effector, PDE5Is stimulate vasodilatation by extending NO’s duration of action [35, 36]. Although their first approved indication was for erectile dysfunction treatment, their therapeutic potential in other pathologies, including acute kidney injury or contrast-induced nephropathy, has recently attracted considerable attention [37]. Kidney disease pathophysiology is associated with renal vasoconstriction. PDE5 inhibitors are thought to protect from kidney injury since PDE5 is highly expressed in the kidney [38], which is consistent with our results.

Reperfusion, i.e., blood flow restoration after ischemia, causes tissue oxidative damage and there are no clear treatment options [39]. Additionally, enhanced cytosolic calcium concentration after rapid oxygen increase during reperfusion may trigger apoptosis [40]. Oxidative stress (reactive oxygen species accumulation) is also considered a main factor in IR injury [41]. NOS has three isozymes (neurogenic NOS, eNOS, and iNOS), and it produces NO [42], which is suggested to mediate IR injury. Large quantities of NO produced by iNOS and eNOS lead to kidney damage, and several studies have proven that high NO concentrations are cytotoxic [43]. Therefore, we analyzed kidney iNOS and eNOS levels. MPO, a heme peroxidase secreted by activated neutrophils, monocytes, and macrophages, is a source of reactive oxygen species [44]. IR and partial nephrectomy increased the levels of iNOS, eNOS, and MPO, and tadalafil significantly protected the kidney from IR injury.

Inflammatory gene analysis revealed that IR and partial nephrectomy induced strong inflammatory responses. Injured tubules and endothelial cells produce TNF-α, IL-1β, and IL-6, the proinflammatory genes induced by lymphocyte activation, because of blocked blood flow and oxygen during ischemia [45]. These proinflammatory factors may result in progressive kidney fibrosis after severe IR injury [46]. Lymphocytes are a key IR injury mediator and they contribute to its pathogenesis [47]. Although lymphocytes are found in tissues after injury, the mechanism involved is unclear [48]. We observed a significant increase in the levels of inflammation-related genes (TNF-α, IL-1β, and IL-6), lymphocytes (CD4 and CD8), neutrophils (MPO), macrophages (F4/80), and monocytes (HLA-DR). Treatment with tadalafil before IR injury protected against elevated inflammation-related gene expression and lymphocyte recruitment in the kidneys.

This study has limitations. First, we did not evaluate tadalafil’s effects in normal Sprague–Dawley rats (without sham operation) or those that underwent left nephrectomy only. Second, a precise comparative analysis of the rats’ ability to recover rapidly was insufficient. Finally, long-term tadalafil effects were not monitored.

## Conclusions

This study demonstrates that the pretreatment of partially nephrectomized Sprague–Dawley rats with tadalafil before IR injury exerts renoprotective effects against inflammation and oxidative stress by reducing the levels of KIM-1, iNOS, eNOS, MPO, and inflammation-related markers (TNF-α, IL-1β, IL-6, CD4, and CD8). Furthermore, treatment with tadalafil preserved renal function by significantly suppressing the loss of viable glomeruli. These findings provide evidence that administering tadalafil before partial nephrectomy may preserve renal function in humans. Large studies are needed to clarify tadalafil’s mechanisms of action.

## Electronic supplementary material

Below is the link to the electronic supplementary material.

Supplementary Material 1

Supplementary Material 2

## Data Availability

No datasets were generated or analysed during the current study.

## Abbreviations

BUN: Blood urea nitrogen
cGMP: Cyclic guanosine monophosphate
eNOS: Endothelial nitric oxide synthase
IR: Ischemia–reperfusion
KIM-1: Kidney injury molecule-1
MPO: Myeloperoxidase
NOS: Nitric oxide synthase
NO: Nitric oxide
PDE-5: Phosphodiesterase type-5
